# HTLV-1 infection interferes with immune responses to Mycobacterium tuberculosis antigens

**DOI:** 10.1186/1742-4690-12-S1-O24

**Published:** 2015-08-28

**Authors:** Natália B Carvalho, Maria de Lourdes Bastos, Yuri Neves, Anselmo Souza, Eduardo Martins Netto, Silvane Santos, Edgar M Carvalho

**Affiliations:** 1 Serviço de Imunologia do Complexo Hospitalar Universitário Professor Edgard Santos (Com-HUPES), Universidade Federal da Bahia (UFBA), Salvador-BA, Brasil; 2 Laboratório de Pesquisa em Infectologia, Com-HUPES, UFBA, Salvador-BA, Brasil; 3 Instituto Brasileiro para a Investigação da Tuberculose/FJS, Salvador-BA, Brasil; 4 Departamento de Ciências Biológicas, Universidade Estadual de Feira de Santana, Feira de Santana-BA, Brasil

## 

Tuberculosis (TB) is still a major health problem. IFN-γ and TNF are critical cytokines in the control of Mycobacterium tuberculosis (Mtb) infection. It has already been demonstrated that human T cell lymphotropic virus type 1 (HTLV-1) infection leads to a spontaneous IFN- γ and TNF production, however HTLV-1 infected subjects possess a 2-4 fold increased risk of developing tuberculosis. Nevertheless, the immune mechanisms involved in this phenomenon are still unclear. The aim of this study was to evaluate immunological features of the association between HTLV-1/Mtb in order to better understand the events leading to higher susceptibility to TB observed in HTLV-1 infected subjects. This was a cross-sectional study evaluating four groups: healthy control (HC group), HTLV-1 infected subjects without TB (HTLV-1 group) or with TB (HTLV-1 + TB group), and individuals with only TB (TB group). TNF, IL-1β, and IL-17 levels were measured in supernatants of non-stimulated or PPD stimulated peripheral blood mononuclear cells (PBMCs) by ELISA. PBMCs from HTLV-1 infected individuals had a decrease (p<0.05) in TNF production following PPD stimulation (median 114.5 pg/ml, IQ range 47-189 pg/ml) compared to non-stimulated cells (202 pg/ml, IQ range 100-250 pg/ml). When we normalized the data to non-stimulated cells, IL-1β production following PPD stimulation by individuals in the HTLV-1 + TB group (3 pg/ml, IQ range 0-43 pg/ml) was lower (p<0.05) than in patients with TB (87 pg/ml, IQ range 14-151 pg/ml). Similarly, HTLV-1/Mtb co-infected individuals had lower (p<0.05) production of IL-17 (9 pg/ml, IQ range 0-41 pg/ml) when compared to TB patients (43 pg/ml, IQ range 13-150 pg/ml). Impairment in TNF, IL-1β, and IL-17 production upon stimulation with mycobacterial antigens may contribute to the higher susceptibility to Mtb infection observed in HTLV-1 infected subjects. Financial support: CNPq, CAPES.

